# Antibody-Dependent Cellular Cytotoxicity and NK Cell-Driven Immune Escape in HIV Infection: Implications for HIV Vaccine Development

**DOI:** 10.1155/2012/637208

**Published:** 2012-04-29

**Authors:** Gamze Isitman, Ivan Stratov, Stephen J. Kent

**Affiliations:** Department of Microbiology and Immunology, University of Melbourne, Melbourne, VIC 3010, Australia

## Abstract

The HIV-1 genome is malleable and a difficult target tot vaccinate against. It has long been recognised that cytotoxic T lymphocytes and neutralising antibodies readily select for immune escape HIV variants. It is now also clear that NK cells can also select for immune escape. NK cells force immune escape through both direct Killer-immunoglobulin-like receptor (KIR)-mediated killing as well as through facilitating antibody-dependent cellular cytotoxicity (ADCC). These newer finding suggest NK cells and ADCC responses apply significant pressure to the virus. There is an opportunity to harness these immune responses in the design of more effective HIV vaccines.

## 1. Introduction

The human immunodeficiency virus (HIV-1) pandemic is causing substantial morbidity and mortality across the globe, particularly in developing countries. Antiretroviral drug therapy for HIV is highly effective in controlling disease; however, eradication of HIV-1 is currently not feasible so treatment is life long and is both expensive and leads to considerable toxicity and drug resistance. A vaccine is widely viewed as being essential to controlling the epidemic. Several advanced efforts to develop an effective vaccine have failed or shown only marginal efficacy to date [1–4]. One of the greatest challenges in developing a vaccine against HIV is to overcome its ability to constantly mutate and escape anti-HIV immune responses. This high mutation rate is a direct result of the presence of the virus' low fidelity RNA polymerase enzyme as well as the high levels of recombination it undergoes [5, 6].

A measure of the pressure immune responses apply is through their ability to force viral mutations that result in escape from immune recognition. Both CTLs and Nabs have long been reported to select for immune escape variants during the course of HIV-1 infection [7, 8]. Much effort in vaccine development centers on inducing broad and potent CTL (cytotoxic T lymphocyte) and Nab (Neutralizing antibody) responses to conserved viral epitopes and restricting opportunities for viral escape. However, it is now also recognised other immune responses, such as antibody-dependent cellular cytotoxicity (ADCC) and NK cells, select for immune escape variants, suggesting additional immune responses apply significant pressure to the virus [9]. ADCC responses mediated by effector NK cells may be useful responses to induce by vaccination. This paper summarizes current thinking on immune escape from anti-HIV immune responses.

## 2. CTL Escape and the Road to Reduced Viral Fitness

Immune escape from HIV was first demonstrated for CTL-based immunity in 1991 [8]. Considerable work since then has shown CTL escape is typically regulated by the effect of the escape mutation on comparative viral fitness, a complex parameter illustrating the overall contribution of all mutation-related advantages and losses (Table 1). Even though the evasion of immune responses presented by escape mutations presents a definite fitness benefit to the virus, the HIV-1 proteome is not infinitely malleable hence the same mutations can result in fitness costs. Some CTL immune escape variants have reduced replicative capacity of the virus (reduced “fitness”) that slows the progression of disease [10, 11]. Studies have demonstrated that certain viruses composed of immune escape mutations are associated with lower viral loads within subjects [12, 13]. It has also been suggested that the rate of viral escape likely reflects the strength of the immune pressure and the fitness cost of the mutant virus [14]. Fitness costs are most dramatically illustrated *in vivo* by the reversion of transmitted escape mutations during acute and early HIV-1 infection [15–19].

Several CTL escape mutations have been confirmed to disrupt normal virus protein structure and/or function [20–23]. More than half of deleterious escape mutations have been verified in the relatively conserved Gag protein, whereas Troyer et al. [24] recently presented that CTL escape mutations in Env did not commonly transfer an associated fitness cost and indeed in a number of cases strengthened competitive viral fitness. This result is consistent with the lack of reversion of Env CTL escape mutations *in vivo* [25, 26]. Macaque and human studies have also demonstrated that escape from T-cell immunity leads to ongoing HIV or SIV infection [27]. The latest investigation of the effect on viral replication of twenty CTL escape mutations in Gag epitopes established only three escape mutations that resulted in substantial reductions in viral replication capacity, indicating that high-cost escape mutations are rather rare [28]. More importantly, these three highly effective CTL escape mutations appeared in epitopes primarily targeted during acute infection by protective HLA class I alleles [29]. This demonstrates that the protection allowed by certain HLA class I alleles may arise because the barrier to viral escape in the targeted epitope is high leading to either maintenance of a dominant and effective CD8+ T-cell response, and/or attenuation of virus replication from selection of high-cost escape mutations. Examination of viruses derived from HIV-1 controllers (individuals who maintain long-term control of HIV-1 viremia) reveal evidence of a role for rare or novel CTL escape-associated fitness costs in control of HIV-1 replication [30–33].

CTL responses and the immune escape variants induced are also important in HIV transmission scenarios. Two recent reports detail early clinical correlates related with the transmission of viruses expressing a number of CTL escape mutations known to weaken *in vitro* replication capacity [12, 34]. As the transmitted escape mutations revert to wild type [23], these enhanced results associated with such transmissions have been perceived to decline and the long-term clinical outcome of these transient effects remains unforeseeable. With improved characterization of the virology of acute HIV-1 infection differences in founder virus, replication compared with viral escape strains caused by dominant CD8+ T-cell responses is becoming easier to model. The distinctions between the founder virus and viral escape strains may contribute to observed variability in the immune control of HIV-1 replication, which may be caused by carry-over mutations and variation in the rate of escape related to fitness costs from key CD8+ T-cell responses, in return influencing set-point viral load and early clinical disease course [35]. Elite control of viral replication may in part be due to the transmission of a virus attenuated by accumulated carryover mutations from hosts with such principal CD8+ T-cell responses to escape associated epitopes resulting in high fitness costs. Rolland et al. recently illustrated the first evidence of selective pressure from vaccine-induced T-cell responses on HIV-1 infection by analyzing HIV-1 genome sequences from 68 volunteers who participated in the STEP Adenovirus-vector HIV-1 vaccine efficacy trial [36]. Comparison of T-cell epitopes in the founder sequences to epitopes in the vaccine distinguished greater breadth for sequences from vaccine recipients than from placebo recipients, suggesting the vaccine imparted important immune pressure to the selection of the infecting isolates. Vaccine-induced fitness-impaired virus could, if sufficiently potent, translate into a reduction in viral loads and attenuation of disease progression.

## 3. The Great Escape from Neutralizing Antibodies

Considerable data exist illustrating the effect of neutralizing antibodies in protecting against HIV-1 infection *in vitro *[37, 38] and *in vivo *using animal models [39–46]. Although antibodies are made to all HIV proteins within a few weeks, only those to the envelope glycoproteins can prevent or neutralize HIV infection. These neutralizing antibodies (Nab) take considerably longer to develop than binding antibodies, generally months to years [47]. HIV-infected subjects almost always develop Nab to their own virus (autologous neutralization), although Nabs typically respond to earlier viral isolates, with the subject's contemporaneous virus having escaped. Some subjects eventually develop Nabs able to cross-neutralize additional viruses (heterologous neutralization), but their concurrent virus is still usually escaped from their autologous Nab. This highlights many of the difficulties involved in controlling HIV replication by Nab and the ability of HIV to escape antibody pressure through a process of genetic change [38]. The envelope gene presents the highest ratio of genetic diversity, most likely as a direct result of Nab pressure. However, for the virus to remain infective, portions of the envelope gene that encode regions essential for functional activity, such as CD4 and coreceptor binding, need to be conserved, and hence escape from Env Nabs probably results in little fitness cost. Individuals who do develop outstanding Nab responses generally have antibodies directed towards such crucial functional regions [48]. Long-term nonprogressors who have remained symptom-free for many years without antiretroviral therapy in general have broader and more potent responses compared to persons who show progressive disease [49–53].

Escape from neutralizing antibody responses often involves serial changes in glycosylation patterns and small insertions and deletions [7]. Richman and colleagues found that 9 of 12 untreated patients with detectable neutralizing antibody had the highest neutralising antibody titer towards against the baseline virus (month 0) whereas only three subjects showed higher titers of neutralizing antibody against viruses that appeared later in infection [7]. Wei and colleagues clearly illustrated the inhibition of HIV-1 by Nabs when successive populations of resistant virus were completely substituted by neutralization-sensitive virus [54]. Furthermore, they showed escape virus contained infrequent mutations in the *env* gene, generally mapped to unknown neutralization epitopes, and involved changes mainly in N-linked glycosylation sites. This pattern of escape led to the hypothesis of an evolving “glycan shield” mechanism of neutralization escape which selected differences in glycan packing preventing Nab binding but not receptor binding. Mutational substitution assays showed that Nab-selected alterations in glycosylation presented escape from both autologous antibody and epitope-specific monoclonal antibodies. Thus a new mechanism was presented contributing to HIV-1 persistence in the presence of an antibody repertoire.

Viral escape regardless of the presence of neutralizing antibodies could demonstrate either that antibodies were ineffective *in vivo*, in which case antibody-sensitive viral strains would remain, or otherwise that the virus escaped the pressure applied by the antibody. Trkola et al. illustrated that passive transfer of Nabs in humans with established HIV resulted in immune escape by comparing the inhibitory activity of 3 monoclonal Nabs (2F5, 4E10 and 2G12) against virus isolates derived before the passive transfer trial and to sequential isolates after antibody treatment [55]. There was a strong association between development of 2G12-resistant viral strains and emergence of escape mutants to this antibody, failure to respond to treatment and loss of viremia control. While evidence of virus escape implies Nab selective pressure to a certain extent [7, 54, 55], it has been speculated that postinfection Nabs could exert only a limited suppressive effect on primary HIV replication [45, 56, 57]. Prevention of primary SIV or SHIV replication in monkeys by passive Nab immunization prior to or very early after infection is achievable [40, 58–60]. Taken together, this suggests that HIV control by potent Nabs is most likely to be effective prior to infection. Immune escape is likely to compromise the role of Nabs after infection is already manifested.

The role of neutralizing antibodies in preventing or limiting HIV-1 infection is becoming clearer with a better understanding of the structure of the envelope glycoprotein as well as passive immunization studies in animals showing that antibodies can indeed control infection. Further insights into neutralization-sensitive epitopes on the envelope glycoprotein are needed that will enable us to design better vaccine immunogens in vaccines. Ultimately this should allow the ability to induce neutralizing antibodies in conjunction with additional antibody-mediated protective mechanisms such as antibody-dependent cell-mediated cytotoxicity (ADCC) in the fight against HIV.

## 4. Escape from ADCC

**Figure 1:**
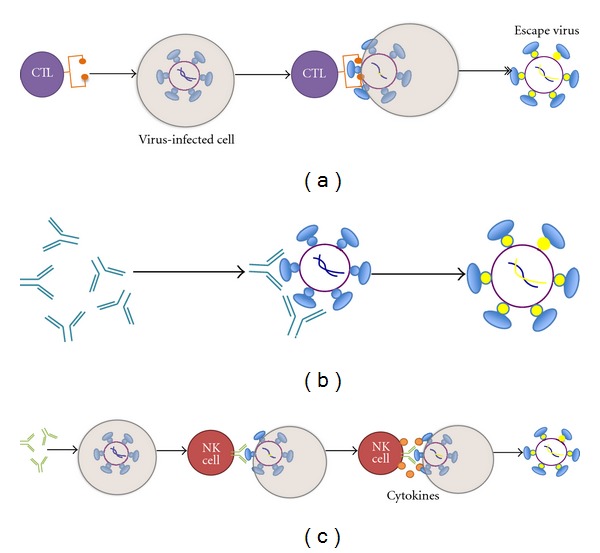
HIV-specific immune responses force immune escape. The mechanism of immune pressure applied by Cytotoxic T lymphocytes (a), neutralizing antibodies (b), and ADCC antibodies (1) is illustrated. Escape from immune responses shows results once free virus (Nab responses) or viral particles are presented either via the MHC class pathway (CTL responses) or possibly on the surface of the infected cell by virus budding (ADCC).

Sequencing single HIV genomes from subjects with acute HIV-1 infection reveals that multiple mutations are acquired during the first months of infection and most align with sites of CTL or Nab escape mutations [61, 62]. However, some mutations do not clearly map to known sites of CTL or Nab escape, suggesting there may be other immune responses, such as ADCC responses, sufficiently potent to select immune escape strains. ADCC antibodies bind to viral antigens on the surface of infected cells and engage Fc receptors on innate immune cells such as NK cells, macrophages, and neutrophils, which in turn lyse the HIV-infected cell (Figure 1(c)).

ADCC is an area relatively poorly explored in HIV immunology in recent years. Very few ADCC epitopes have been identified to date within HIV. The majority of these identified ADCC epitopes are within Env glycoproteins, gp120 [63–66] and gp41 [67–69], Tat [70] and Nef [71, 72].

Several recent studies in both humans and macaques are now suggesting that ADCC antibodies can be effective in controlling HIV or SIV [4, 73, 74]. The potent immune pressure that can be applied by HIV-specific ADCC antibodies has only recently been brought into sharper focus. Importantly, Hessell and colleagues performed experiments on Nab mutated in the Fc region, which showed a reduced ability of the Nab to mediate killing of infected cells *in vitro*. When they administered the mutant (Fc defective) Nab to macaques, they were also markedly reduced in their ability to prevent SHIV infection [73]. It perhaps should be no surprise that ADCC antibodies are also implicated in viral escape as the HIV genome is able to make multiple changes to avoid CTL, Nab, and antiretroviral drug pressure.

ADCC responses forcing immune escape had until recently not previously been demonstrated. Demonstrating viral escape from ADCC responses would strongly suggest that ADCC responses exert significant pressure on the immune system [75]. Stratov et al. described a novel assay which allowed the mapping of a series of HIV-specific ADCC epitopes in subjects infected with HIV, using a set of consensus HIV peptides [76]. The identified epitopes within the subjects HIV strains were then further cloned and sequenced across the relevant epitopes and analyzed as to whether their ADCC responses were able to recognize their own virus strain. Evidence of immune escape was found against multiple HIV-specific ADCC epitopes studied in the Env protein of HIV-1 [9]. Evolution of escape over time was detected in contemporary plasma samples, which differed significantly from initial viral sequences at areas targeted by ADCC.

ADCC antibody responses are generally thought to target viral surface proteins presented on the surface of infected cells. Our group has also identified ADCC responses to viral peptides derived from internal HIV-1 proteins such as Vpu and Pol [75, 76]. Interestingly, we also identified possible immune escape to an epitope of the highly conserved protein Pol [77]. It is not immediately apparent how these epitopes would be presented on the surface of cells to ADCC antibodies and force viral escape and much more work needs to be done to define whether ADCC antibodies to internal proteins can recognize HIV-infected cells *in vitro*. We speculate that it may also be possible that ADCC recognition of viral debris on the surface of healthy neighboring cells may trigger noncytolytic activity from NK cells that could limit HIV-1 spread in a local environment. Such a mechanism would also be susceptible to immune escape.

Using ADCC peptide epitopes and an NK-cell activation assay, the hypothesis that ADCC plays a major part in the immune response against HIV was confirmed. This work likely underestimates the number of ADCC epitopes targeted by each HIV+ subject, since linear epitopes are readily mapped and dissected and consensus B overlapping peptides are used for screening. Conformational ADCC antibodies are likely to elicit escape also but to map such responses and identify escape patterns will be more difficult and require large numbers of mutant whole Env proteins.

The partial efficacy shown by the recently reported canarypox prime/protein boost vaccine trial conducted in Thailand [78] could possibly be associated with ADCC antibodies. Recent conference presentations have correlated nonneutralizing antibody responses to vaccine efficacy, although much work remains to be done to understand this fully [79]. It is conceivable, by analogy with results on CTL responses in the STEP trial [36], that vaccinated subjects in the Thai trial who still became infected may have become infected with HIV variants already “preescaped” at the ADCC responses induced by their vaccination.

ADCC-forced mutations could theoretically incur some “fitness cost” to viral replicative capacity, similar to that observed for CTL escape variants [12]. Constructing replicating viruses with ADCC-induced mutations will allow testing of this hypothesis. More potent ADCC antibodies are likely to target conserved or functional domains of viral proteins. The Env protein is highly diverse and readily escapes CTL and Nab responses with apparent minimal fitness costs [7, 24, 26, 80]. It is possible that any fitness cost of ADCC escape in Env could also be small. ADCC antibodies targeting conserved non-Env proteins such as Vpu and Pol may be more potent, although it needs to be assessed whether these antibodies recognize HIV-infected cells as noted above. It is also likely that compensatory mutations may emerge which repair any fitness cost of primary mutations [81]. Further studies on the patterns of ADCC escape and the specific cellular components involved in ADCC should allow a finer understanding of how to either limit ADCC escape or force larger fitness costs.

Natural killer (NK) cells are the key effector cells mediating ADCC function. Virally infected cells are identified through a range of activating and inhibitory receptors [82] as well as both activating and inhibitory killer immunoglobulin-like receptors (KIRs) [83, 84]. Alter and colleagues recently clearly demonstrated that NK cells can directly mediate antiviral immune pressure *in vivo* in humans [85]. They showed that the binding of inhibitory KIRs to HIV-1-infected CD4+ T cells is amplified and the antiviral activity of KIR-positive NK cells is diminished by KIR-associated HIV-1 sequence polymorphisms. Similar to immune pressure applied by virus-specific T cells and neutralizing antibodies, it seems plausible to state that KIR-positive NK cells can place immunological pressure on HIV-1 and that the virus can evade such NK-cell-mediated immune pressure by selecting for sequence polymorphisms.

## 5. Conclusions

CTL and Nab immune responses are pivotal drivers in immune escape and viral variability. It is now clear that the role of NK cells in viral selection, both through direct killing and ADCC mechanisms, is likely to have been previously underestimated. Other effector cells of the innate immune system, including macrophages and neutrophils, may also be important in driving HIV evolution. Evidence of the pressure applied by ADCC antibodies now provides challenges to inducing the most effective ADCC antibodies by vaccination. A better understanding of the immune responses to HIV is required to fully harness the potential of a vaccine to both prevent viral entry and ongoing infection.

## Figures and Tables

**Table 1:** Key escape papers.

Immune response	Hypothesis	Result	Ref.
CTL based	HLA-B*57/B*5801 CTL escape mutations in Gag impacts viral replication *in vivo *	Reductions in relative replication capacity reduce “viral fitness”	[20]
	CTL escape mutations in Env do not result in reduced viral fitness	Escape mutations within Env-specific CTL are epitopes evident but no correlation with reduced SIV replication	[25]
	Step HIV-1 vaccine trial exerts selective CTL pressure on HIV-1	Extended sequence divergence for vaccine recipients who become infected suggests vaccine-induced CTL imparted significant immune pressure Gag-84 most significant signature site	[36]

Nab based	Evolving “glycan shield” mechanism prevents Nab binding	Env gene mutations in escape virus sparse Escape mutations did not map to known epitopes Efficient neutralization requires potent, high titres	[54]
	Continual selection of Nab escape variants occurs	All previous viral isolates, but not concurrent isolate, are recognised by concurrent Nab	[7]
	Passive transfer of human neutralizing monoclional antibodies delays HIV-1 rebound post-antiretroviral therapy	2G12 monoclonal was crucial for transient *in vivo* effect of Nab cocktail but immune escape resulted	[55]

ADCC based	Immune pressure from HIV-specific ADCC results in immune-escape variants	ADCC causes escape in multiple epitopes and evolves over timeADCC antibodies forcing immune escape can be non-eutralizing	[9]
	NK cells apply immunological pressure on HIV-1 through direct killing of infected cells	HIV-1 selects KIR2DL2+ virus mutations that result in reduced antiviral activity of NK cells	[85]
